# Macrolide antibiotics activate the integrated stress response and promote tumor proliferation

**DOI:** 10.15698/cst2023.04.278

**Published:** 2023-03-21

**Authors:** Xin Yu, Ai-Ling Tian, Ping Wang, Juanjuan Li, Juan Wu, Bei Li, Zhou Liu, Siqing Liu, Zhijie Gao, Si Sun, Shengrong Sun, Yi Tu, Qi Wu

**Affiliations:** 1 Department of Breast and Thyroid Surgery, Renmin Hospital of Wuhan University, Wuhan, Hubei, P. R. China.; 2 Gustave Roussy Cancer Campus, Villejuif Cedex, France.; 3 Université Paris-Saclay, Faculté de Médecine, Le Kremlin-Bicêtre, France.; 4 Centre de Recherche des Cordeliers, INSERM U1138, Équipe Labellisée - Ligue Nationale contre le Cancer, Université Paris Cité, Sorbonne Université, Paris, France.; 5 Metabolomics and Cell Biology Platforms, Gustave Roussy Cancer Campus, Villejuif, France.; 6 Medical College, Anhui University of Science and Technology, Huainan, AnHui, P. R. China.; 7 Department of Pathology, Renmin Hospital of Wuhan University, Wuhan, Hubei, P. R. China.; 8 Department of Clinical Laboratory, Renmin Hospital of Wuhan University, Wuhan, Hubei, P. R. China.; 9 Tongji University Cancer Center, Shanghai Tenth People's Hospital, School of Medicine, Tongji University, Shanghai, P. R. China.

**Keywords:** macrolide antibiotic, azithromycin, autophagy, ROS, ER stress, cancer

## Abstract

Macrolide antibiotics are widely used antibacterial agents that are associated with autophagy inhibition. This study aimed to investigate the association between macrolide antibiotics and malignant tumors, as well as the effect on autophagy, reactive oxygen species (ROS) accumulation and integrated stress response (ISR). The meta-analysis indicated a modestly higher risk of cancer in macrolide antibiotic ever-users compared to non-users. Further experiments showed that macrolides block autophagic flux by inhibiting lysosomal acidification. Additionally, azithromycin, a representative macrolide antibiotic, induced the accumulation of ROS, and stimulated the ISR and the activation of transcription factor EB (TFEB) and TFE3 in a ROS-dependent manner. Finally, animal experiments confirmed that azithromycin promoted tumor progression *in vivo*, which could be receded by N-acetylcysteine, an inhibitor of ROS and ISR. Overall, this study reveals the potential role of macrolide antibiotics in malignant progression and highlights the need for further investigation into their effects.

## INTRODUCTION

As one of the most extensively applicable agents, the use of antibiotics has emerged to bring questionable advances in the therapeutic strategies of patients with cancer. Increasing evidence indicates that long-term or inappropriate antibiotic abuse is linked to an increased risk of cancer [1-4]. In addition, the overuse of antibiotic therapy is attributable to resistance to chemotherapy and immunotherapy [5-7]. However, targeted antimicrobial therapy can reduce the incidence and mortality of gastric cancer caused by *Helicobacter pylori*, which is a well-known risk factor in stomach cancer [8]. Therefore, the rational use of antibiotics in patients with cancer needs further exploration.

Macrolides are compounds that are composed of macrocyclic lactone rings, and are extensively used as antibiotics and immune inhibitors. As a category of antibiotics, they strictly consist of 14-, 15-, or 16-membered lactone rings [9, 10]. For example, erythromycin and roxithromycin (RA) possess the structure of 14-lactone rings, azithromycin (AZD) belongs to the family of 15-lactone rings, and the representative agent consisting of 16-lactone rings is spiramycin (SP) [9]. Additionally, tacrolimus and sirolimus possessing of macrolide amide structures have been used as immunosuppressants or immunomodulators after liver or renal transplantation and for treatment of autoimmune disease [11, 12]. In clinical practice, macrolide antibiotics are first-line drugs for typical community-acquired pneumonia and chlamydia infection. Moreover, macrolide antibiotics, including brefeldin A and AZD, exert considerable antitumour effects both *in vitro* and *in vivo* [13-15]. Likewise, macrolide antibiotics could restore the sensitivity of tumor cells to agents, bortezomib, CDK4/6 inhibitors, or EGFR-TKIs [16-19]. However, there is evidence suggesting that the prophylactic use of AZD can increase the incidence of haematological relaxation by 70% two years after haematopoietic cell transplantation [20]. Besides, exposure to AZD is associated with an increased risk of subsequent neoplasm after hematopoietic cell transplantation [21]. Therefore, the potential role of macrolide antibiotics in malignant progression requires further exploration.

The integrated stress response (ISR) is characterized by the phosphorylation of the eukaryotic initiation factor 2α (eIF2α) [22]. As a phylogenetically conserved protein, eIF2α can be phosphorylated by four main kinases: heme-regulated inhibitors, general control nonderepressible-2, protein kinase double-stranded RNA-dependent (PKR) and PKR-like endoplasmic reticulum (ER) kinase (PERK) [23]. ISR has a pivotal effect on the adaptation to oxidative stress or ER stress, initiation of autophagy, mitochondrial homeostasis, and innate cellular defense against viral infections [22, 24-26]. However, ISR has been shown to promote the growth and metastasis of malignant tumors by inducing epithelial-mesenchymal transition (EMT) and angiogenesis [27]. Moreover, the crucial lysosomal transcription factors, transcription factor EB (TFEB) and TFE3, have been found to mediate the cellular response to ISR [28]. PERK and phosphorylated eIF2α activate TFEB and TFE3 after treatment with lysosomotropic agents (such as AZD) and ER stressors (such as thapsigargin (TG) and tunicamycin (TM)) [28, 29]. Macrolides such as AZD are known to be trapped in lysosomes by enhancing protonation under acidic conditions, leading to lysosomal acidification dysfunction and lysosomal function blockage via increases in their concentration in the lysosome until lysosomal membranes are destabilized [30, 31]. Our previous results indicate that lysosomotropic agents including 3-hydroxychloroquine, chloroquine, and AZD, have the capacity to stimulate ISR by blocking autophagic flux, resulting in the activation of TFEB and TFE3 [29]. Potentially, macrolide antibiotics may affect malignant biological properties by inducing ISR.

In this work, macrolide antibiotics were identified as potent autophagy inhibitors that induce a loss of lysosomal acidification. Furthermore, the representative agent, AZD, induced the accumulation of intracellular reactive oxygen species (ROS) and stimulated ISR, leading to the activation of TFEB and TFE3. Ultimately, treatment with AZD accelerated tumor growth.

## RESULTS

### Meta-analysis indicated a correlation between the use of macrolide antibiotics and an increased risk of malignant tumors

Previous studies have suggested that the overuse of antibiotics can increase the risk of cancer and reduce the efficacy of chemotherapy and immunotherapy [1-3, 5-7]. To further investigate the association of macrolide antibiotic use with tumors, we retrospectively searched and identified 36,578 publications. Ten studies, including 825,068 individuals and over 154,760 malignant tumor cases, met the eligibility criteria (Fig. S1, Table S1) [32-41]. The studies included were published in English from 2008 to 2015. Three studies were conducted in Canada [35, 37, 38], three in the UK [34, 39, 40], one in the USA [32], one in Denmark [33] and one in the Netherlands [41]. Studies included various cancer types, such as breast, lung, and colorectal cancer, etc.

**Figure 1:**
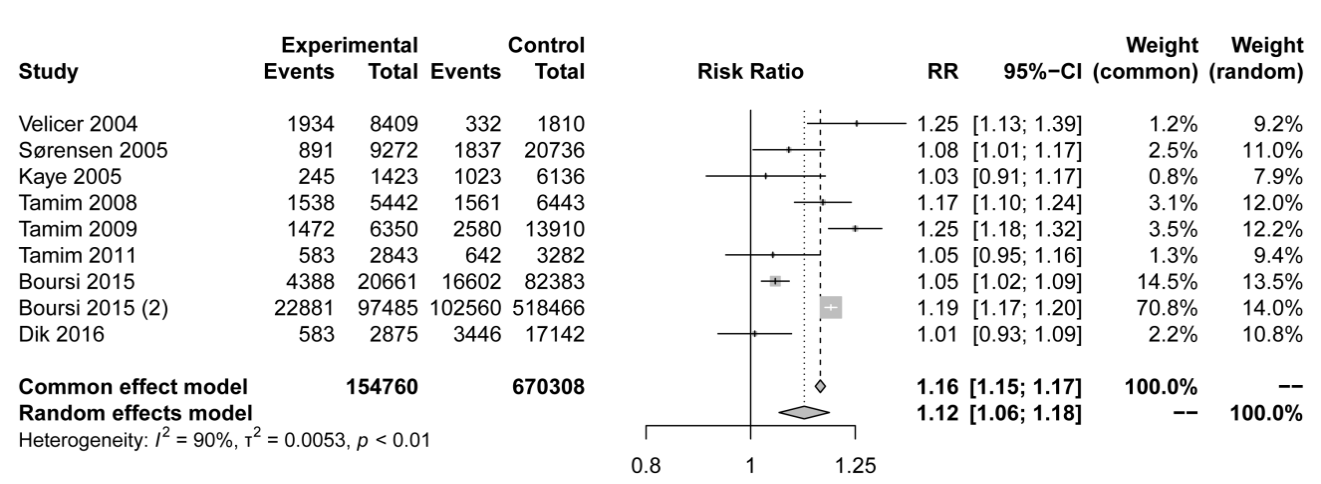
FIGURE 1: Forest plot examining the association between macrolide antibiotic ever-use versus nonuse and the risk of cancer.

The pooled risk estimate indicated that compared to non-users, macrolide antibiotic ever-users had a modestly higher risk of cancer (RR=1.12,95% CI 1.06-1.18, **Fig. 1**). Although the meta-analysis showed statistical heterogeneity among the studies (I^2^ = 90%), no evidence indicated a significant influence in any single study (Fig. S1A). Asymmetrical towards positive associations was not presented in the funnel plot (Fig. S1B), revealing no significant publication bias (Egger's test p=0.2467). In conclusion, our meta-analysis showed that the use of macrolide antibiotics was associated with a higher incidence of malignancy, but the mechanism requires further exploration.

### Macrolide antibiotics are potential autophagic inhibitors

**Figure 2:**
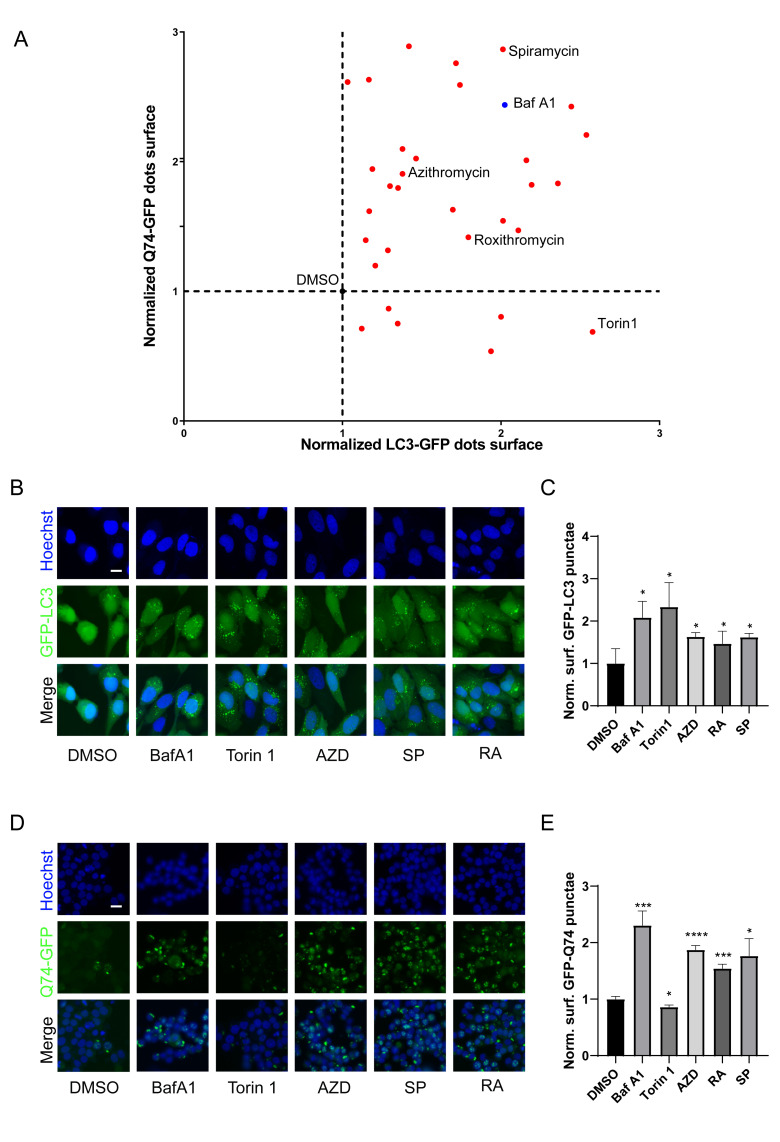
FIGURE 2: Identification of macrolide antibiotic as potent blockers of autophagic flux. **(A)** GFP-LC3B-expression U2OS cells and GFP-Q74-expression PC12 cells were treated with Torin 1 (300 nM), Baf A1 (100 nM) or macrolides (40 µM) for 6 h. **(B, C)** GFP-LC3-expression U2OS cells or **(D, E)** GFP-Q74-expression PC12 cells were treated with torin 1, Baf A1, AZD (40 µM), RA (40 µM) or SP (40 µM) for 6 h. Representative images are presented **(B, D)**. The **(C)** GFP-LC3B or **(E)** GFP-Q74 puncta were assessed. Scale bars equal 10 μm. *P<0.05; **P < 0.01; ***P < 0.001; **** P < 0.0001 compared with DMSO/control.

Macrolides exert their antibacterial effects by interfering with ribosomal function [9], and recent evidence indicates that they can also cause lysosomal acidification disorders [30], which affect the autophagy process. To investigate the influence of macrolides on autophagy, a library of macrolides was screened for their role in autophagic flux through GFP-microtubule-associated proteins 1A/1B light chain 3B (MAP1LC3B/LC3B)-expressing U2OS cells and GFP-Q74-expressing PC12 cells (macroautophagy can be detected by measuring the degradation of GFP that aggregates in the cytoplasm) [42]. Similar to Bafilomycin A1 (Baf A1, lysosomal acidification inhibitor), almost all macrolide antibiotics (except kitasamycin) increased puncta of GFP-LC3B and GFP-Q74, while macrolide immunosuppressants increased GFP-LC3B punta but decreased GFP-Q74 puncta, similar to Torin 1 (mechanistic target of rapamycin inhibitor; **Fig. 2A**, Table S2). The stimulating effects of RA, AZD, and SP on GFP-LC3B and GFP-Q74 dots were verified (**Fig. 2B-E**). In summary, macrolide antibiotics induce the accumulation of LC3B and Q74, and appear to be potential inhibitors of autophagy.

### Macrolide antibiotics block autophagic flux

**Figure 3:**
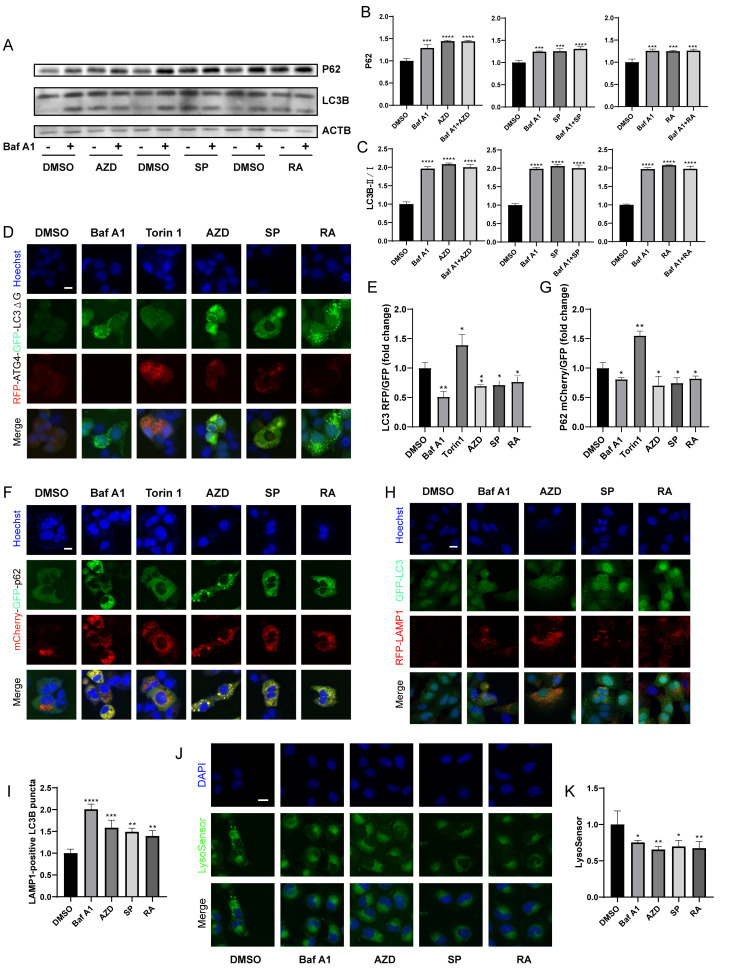
FIGURE 3: Macrolide antibiotics block the autophagy flux. **(A)** U2OS cells were treated with AZD (40 µM), RA (40 µM) or SP (40 µM) combined or not with Baf A1 (10 nM) for 4 h. Then the western blot was conducted. And the relative expression of P62 and LC3B II/I was calculated **(B, C)**. **(D, E)** GFP-ATG4-RFP-LC3BΔG-expression and **(F, G)** mCherry-GFP-p62-expression U2OS cells were treated with Torin 1 (300 nM), Baf A1, AZD, RA or SP for 6 h. The fluorescence of GFP and RFP was measured, and the RFP/GFP ratio was calculated **(E, G)**. **(H, I)** GFP-LC3 and RFP-LAMP1 U2OS cells were treated with Baf A1, AZD, RA and SP for 6 h. And the LAMP1-positive LC3B puncta were measured **(I)**. **(J, K)** U2OS cells were treated with Baf A1, AZD, RA and SP for 6 h, and incubated with LysoSensor for 30 min at 37°C. Then the average intensity of lysoSensor fluorescence was assessed **(K)**. Representative images are presented **(J)**. Scale bars equal 10 μm. *P<0.05; **P < 0.01; ***P < 0.001; **** P < 0.0001 compared with DMSO.

Next, we further determined whether macrolide antibiotics block actual autophagic flux. Immunoblot analyses showed that AZD, RA and SP induced the accumulation of P62 and LC3B (**Fig. 3A-C**), and in combination with Baf A1 increased the accumulation of P62 but not LC3B compared to Baf A1 alone, indicating that macrolides block autophagic flux [43]. Besides, RFP-ATG4-GFP-LC3BΔG-expressing U2OS cells were used. Experiments confirmed that AZD, RA and SP consistently decreased the RFP/GFP ratio of cells, indicating a decrease in autophagic flux (**Fig. 3D, E**) [42]. Similarly, mCherry (pH-resistant)/GFP (pH-sensitive) was reduced in mCherry-GFP-sequestosome 1 (SQSTM1/p62) expressing cells treated with AZD, RA and SP (**Fig. 3F, G**). In addition, AZD, RA and SP induced an increase in LAMP1-positive LC3B puncta (**Fig. 3H-I**), but decreased lysosomal acidity assessed by Lysosensor (**Fig. 3J-K**). These results indicate that macrolide antibiotics block autophagic flux by inhibiting lysosomal acidification without hindering autophagosome-lysosome fusion.

### AZD induces ROS accumulation and endoplasmic reticulum (ER) stress

**Figure 4:**
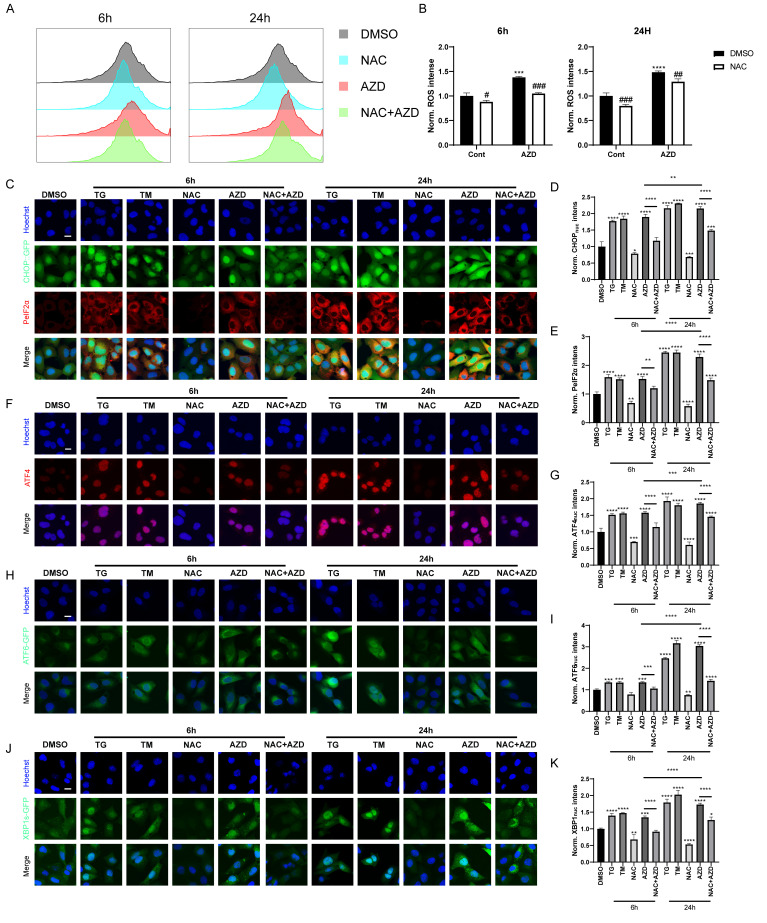
FIGURE 4: AZD induces ROS accumulation and ER stress. **(A, B)** ROS generation was detected by DCFH-DA probe. MCA205 cells were treated with AZD (40 µM) combined or not with NAC (3 mM) for 6 h or 24 h. Fluorescence intensity histogram **(A)** and average fluorescence intensity **(B)** are presented. **(C-E)** CHOP::GFP-expression U2OS cells were treated with TM (3 μM), TG (3 μM), AZD, NAC or NAC combined AZD for 6 h or 24 h, and PeIF2α was assessed by immunostaining. Representative images are presented **(C)**. The intensity of fluorescence was measured **(D, E)**; **(F-K)** wild type, ATF6-GFP-expression and XBP1s-ΔDBD-venus-expression U2OS cells were treated with TM, TG, AZD, NAC or NAC combined AZD for 6 h or 24 h, then ATF4 was assessed by immunostaining, ATF6 and XBP1s was assessed by GFP intensities. Representative images of ATF4 **(F)**, ATF6 **(H)** and XBP1s **(J)** nuclear translocation are presented. The average nuclear intensity of ATF4 **(G)**, ATF6 **(I)** and XBP1s **(K)** was assessed. Scale bars equal 10 μm. *P<0.05; **P < 0.01; ***P < 0.001; **** P < 0.0001 compared with DMSO. And the horizontal line indicates the comparison between the two groups.

When autophagic flux is blocked, misfolded and unfolded proteins accumulate, leading to the unfolded protein response (UPR). In addition, recent studies have shown that macrolide antibiotics, particularly AZD, inhibit mitochondrial function and increase ROS levels [44], which are known inducers of ER stress and autophagy [45, 46]. Therefore, we used AZD as a representative macrolide antibiotic to investigate the role of macrolide antibiotics in ROS and ER stress. Flow cytometry analysis results showed that AZD induced ROS accumulation in MCA 205 cells, which could be reversed by N-acetylcysteine (NAC), a scavenge of ROS (**Fig. 4A-B**) [45]. Furthermore, AZD induced signs of ER stress like TG and TM (ER stress inducing agents; **Fig. 4C–K**, Fig. S2A-E), including upregulation of phosphorylation of eIF2α on serine 51 (PeIF2α) and GFP-C/EBP homologous protein (CHOP) in U2OS cells (**Fig. 4C-E**, Fig. S2A, B). Other markers of ER stress, including the nuclear presence of activating transcription factor 4 (ATF4) (**Fig. 4F, G**, Fig. S2A, C), ATF6 (**Fig. 4H, I**, Fig. S2A, D), and the spliced isoform of X-box binding protein 1 (XBP1s; **Fig. 4J, K**, Fig. S3 A, E) were also induced by AZD. In addition, ER stress induced by AZD could be reversed by NAC (**Fig. 4C–K**, Fig. S2A-E). These results indicate that AZD is a potent ER stressor that induces the phosphorylation of eIF2α and that this induction is at least partially dependent on accumulation of ROS. Additionally, the accumulation of LC3B and P62 induced by AZD was partially suppressed by NAC (Fig. S2F), while the increase in mRNA levels of LC3B and P62 was significantly inhibited (Fig. S2G, H), suggesting that AZD may promote the intracellular accumulation of LC3B and P62 through two pathways, autophagy blockade and induction of cellular stress.

### AZD induces TFEB/TFE3 activation

**Figure 5:**
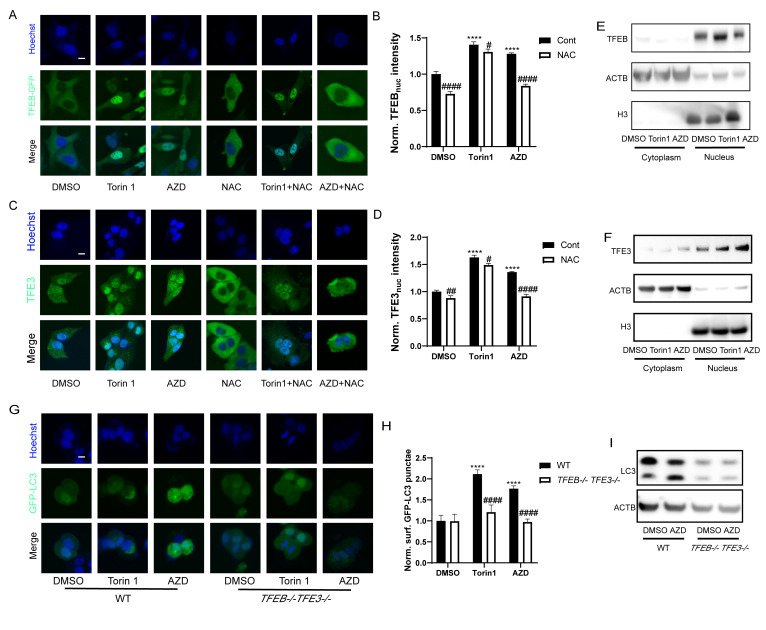
FIGURE 5: AZD induces TFEB/TFE3 activation. **(A-F)** TFEB-GFP-expressing or wild type U2OS cells were treated with Torin 1 (300 nM) or AZD (40 µM) combined or not with NAC (3 mM) for 6 h. The average nuclear intensity of TFEB was measured **(A, B)**, TFE3 translocation was assessed by immunostaining **(C, D)**, and **(E)** TFEB and **(F)** TFE3 in the cytoplasm and nucleus were assessed by Western blot. **(G)** Western blot was performed for measuring the expression of TFEB and TFE3 in U2OS cells with or without *TFEB-/-TFE3-/-* double-knockout treated with AZD. Scale bars equal 10 μm. (*P < 0.05, **P < 0.01, ***P < 0.001,****P<0.0001 vs. DMSO; #P < 0.05, ##P < 0.01, ###P < 0.001, #### P < 0.0001 vs. Cont or WT).

TFEB and TFE3 are transcription factors that act as master genes controlling lysosomal biosynthesis and autophagy. Their activation is triggered by lysosomal dysfunction [47], ER stress, nutrient deficiency, mitochondrial damage and pathogen infection [48]. Given that macrolide antibiotics can influence the lysosome, they also potentially activate TFEB and TFE3. Indeed, nuclear translocation of the TFEB-GFP fusion protein was observed in U2OS cells treated with AZD (**Fig. 5A, B**). Likewise, immunofluorescence results showed that the nuclear translocation of TFE3 was also promoted by AZD (**Fig. 5C, D**). Consistently, immunoblot detection of TFEB and TFE3 in the cytoplasm and nucleus showed similar results (**Fig. 5E, F**). Likewise, the activation of TFEB and TFE3 translocation induced by AZD was inhibited by NAC, while the activation of TFEB and TFE3 induced by Torin1 was limited influenced by NAC (**Fig. 5A, C**, Fig. S3A-C). Moreover, AZD induced the lipidation and puncta of LC3B were impeded by the double knockout of TFEB and TFE3 (genotype: *TFEB−/− TFE3−/−*; **Fig. 5G-I**). In addition, interactions between ER stress and TFEB/TFE3 activation induced by AZD were observed. The translocation of TFE3 and accumulation of LC3B induced by AZD were blunted in USOS with a mutant nonphosphorylation of eIF2α (eIF2α_S51A_; Fig. S3D-F). Likewise, *PERK*^*-/-*^ cells exhibited reduced activation of TFE3 (Fig. S4G, H). The activation of CHOP (Fig. S3I, J) and ATF4 (Fig. S3K, L) was inhibited in *TFEB*^−/−^ *TFE3*^−/−^ cells. These results indicate that activation of TFEB and TFE3 represents a key molecular link between ER stress and autophagy induced by AZD.

### AZD induced ER stress and promoted tumor growth *in vivo*

**Figure 6:**
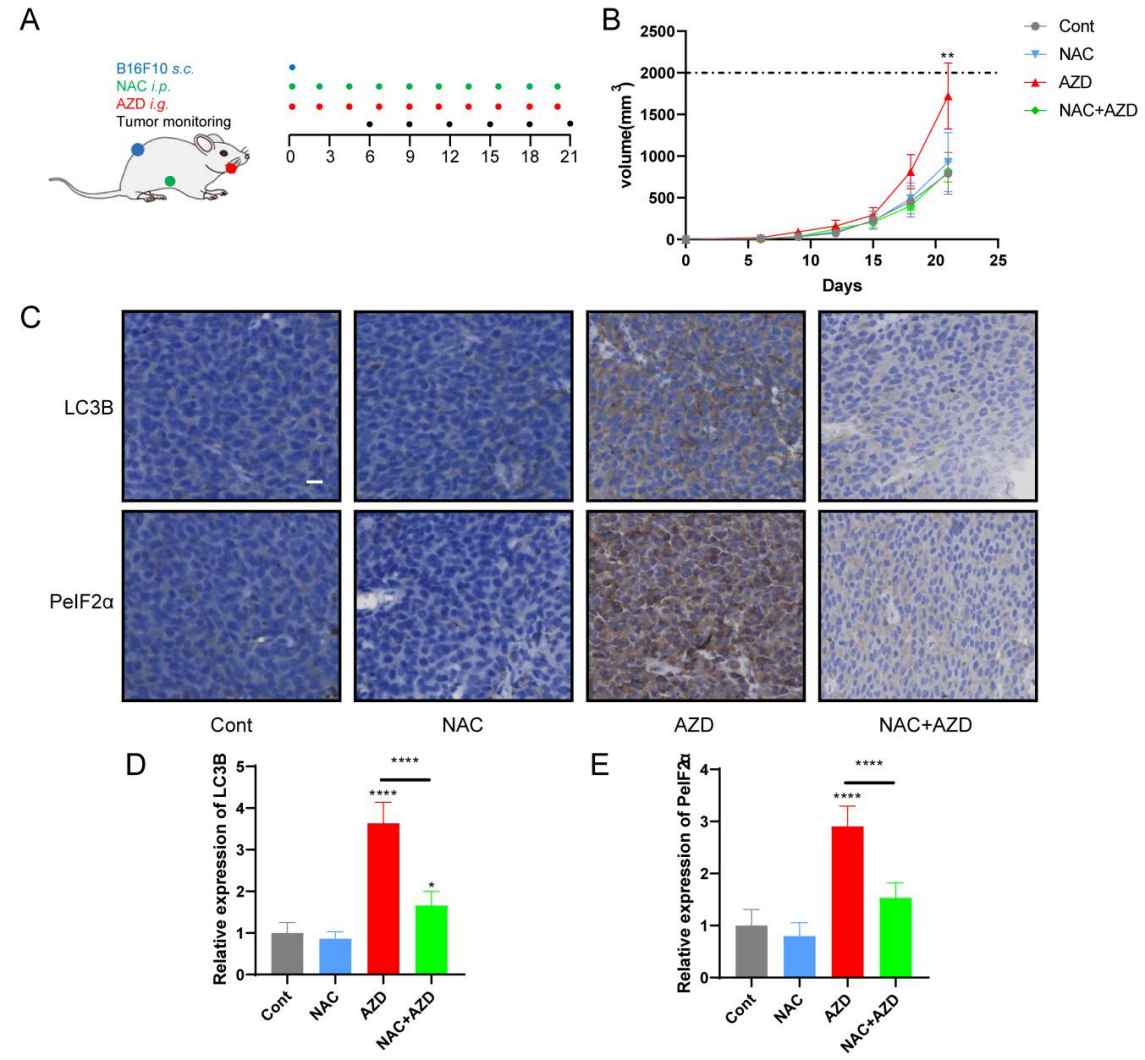
FIGURE 6: NAC reverse AZD-induced tumor growth stimulation. **(A-C)** *In vivo* treatment of implanted murine B16F10 melanoma with AZD combined or not with NAC (schematic view in A). **(B, C)** The data of administration of AZD combined or not with NAC, depicted as **(C)** growth curves (mean±SD). **(D-F)** Immunohistochemical analysis was performed on paraffin-embedded sections of B16F10 melanoma tissue in mice by using LC3B and eIF2α. Representative images are presented **(D)** and the staining score was assessed **(E, F)**. Scale bars equal 20 μm. *P<0.05; **P < 0.01; ***P < 0.001; **** P < 0.0001 compared with Cont. And the horizontal line indicates the comparison between the two groups.

Sustained ER stress results in chronic inflammatory responses, which may contribute to cancer genesis and progression [45, 46]. We determined the capacity of AZD to influence tumor growth *in vivo* by measuring the growth of B16F10 melanoma and MCA205 fibrosarcoma in immunocompetent mice treated with AZD, NAC or a combination (**Fig. 6A**, Fig. S4A). The results indicate that AZD accelerated the progression of tumors (**Fig. 6B**, Fig. S4B, C), increased the accumulation of LC3B, and enhanced the expression of PeIF2α *in vivo* (**Fig 6C-E**), and these effects were attenuated by NAC. These findings indicate that AZD induces ER stress and promotes tumor growth *in vivo.*

## DISCUSSION

**Figure 7:**
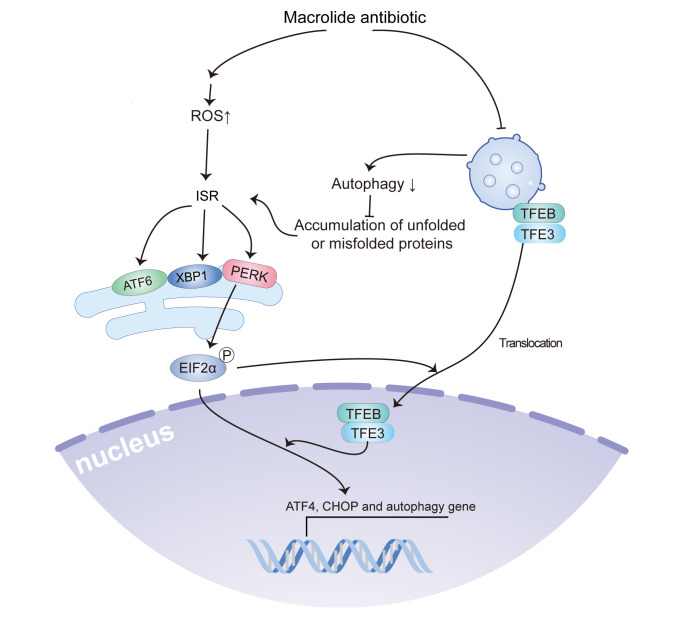
FIGURE 7: Overview of macrolide antibiotics activate the ISR. Macrolide antibiotics inhibit lysosomal acidification, leading to the blockade of autophagic flux and the accumulation of unfolded or misfolded proteins. Moreover, they induce the accumulation of ROS. The combined accumulation of unfolded/misfolded proteins and ROS induces an ISR, including the activation of PeIF2α/ATF4/CHOP signaling, XBP1 splicing, and ATF6 translocation, which in turn contribute to the translocation of TFEB and TFE3. Furthermore, TFEB and TFE3 are involved in the accumulation of LC3B-bound autophagosomes and the activation of ATF4 and CHOP.

The current study indicated that macrolide antibiotics, such as AZD, RA, and SP, could reduce autophagic flux by inhibiting lysosomal acidification, and induced ROS accumulation and ROS accumulation-dependent ER stress, including the activation of PeIF2α/ATF4/CHOP signaling, the splicing of XBP1, and ATF6 translocation, which contribute to the translocation of TFEB and TFE3. Furthermore, TFEB and TFE3 were involved in the accumulation of LC3B-binding autophagosomes and activation of ATF4 and CHOP (**Fig. 7**). Notably, the use of macrolides has been associated with an increased incidence of malignant tumors, and treatment with AZD promotes tumor growth in a preclinical model in a ROS accumulation and ER stress-dependent manner.

Macrolide antibiotics are a commonly used class of antibiotics composed of a large macrocyclic lactone ring. However, their effect on malignant progression is still ambiguous. On one hand, macrolide drugs have been shown to retard tumor growth. For example, AZD treatment is reported to reduce tumor cell proliferation in a dose-dependent manner. Mechanistically, AZD could block autophagy-associated degradation to evaluate the levels of death receptors, and then synergize with the effect of tumor necrosis factor-related apoptosis-inducing ligand on apoptosis induction [15]. On the other hand, macrolides combined with β-lactam^+/−^ inhibitors and fluoroquinolones have been found to accelerate tumor growth and to impair the antitumor effects of immune checkpoint inhibitors (ICIs) [6, 52]. Since the antitumor immune response in the host has increasingly exerted a vital effect on malignant disease, the different results may depend on the disparity of mouse models. Furthermore, in an antibiotic-treated group, there was significantly shorter overall survival and progression-free survival [6, 53].

Recent studies have shown that long-term AZD treatment is capable of increasing the levels of ROS and releasing ROS into diverse tissues and organs [57]. At mild concentrations, ROS can stimulate oncogenesis and support tumor proliferation[58]. The evidence suggests that AZD can promote ROS accumulation by inducing mitochondrial dysfunction at doses that do not cause autophagy blockade [44]. Considering that autophagy blockade is also a trigger for ROS accumulation [59], AZD may induce ROS accumulation through multiple pathways. ISR is remarked upon by eIF2α phosphorylation and activation of its downstream pathways is required for initiation of autophagy, UPR, redox homeostasis, and defense against viral infections [22]. Moderate ISR can facilitate tumor growth and metastasis by promoting EMT, tumor cell dormancy, and tumor initiating cell function, as well as supporting tumor immune evasion and angiogenesis [27]. Recent studies have shown that AZD, as autophagy blocker, stimulates ISR in infectious diseases, neurogenic disorders and cancer [29, 54-56]. Likewise, activation of eIF2α and TFEB endows cancer cells with the capacity to increase antioxidant transcription factors and generate antioxidant substances, which assists tumor cells to maintain oxidant-antioxidant homeostasis [28, 60]. Thus, AZD may promote tumorigenesis by inducing a moderate increase in ROS levels. Ultimately, the homeostasis of gut microbiota has emerged as a key factor that impacts tumorigenesis and the tumor-specific immune response [52, 61]. Antibiotic therapy, such as macrolide application, markedly reduces the diversity of gut microbiota, and specifically eliminates some beneficial commensal bacteria, consequently leading to a loss of protective functions for the host. Hence, further fundamental studies and intense clinical trials are required to develop multiple approaches containing selective antibiotic therapy or probiotic transplantation. Currently, AZD is widely considered a safe antibiotic by most practitioners and is commonly used to treat various bacterial infections. Based on the results of this study, the potential risks and benefits for cancer patients should be carefully evaluated to determine whether AZD prescriptions should be issued.

In conclusion, this study provides evidence that macrolide antibiotics may contribute to malignant progression by inhibiting autophagy, inducing ROS accumulation, and activating ISR. The potential role of macrolide antibiotics in cancer development and progression should be taken into consideration in their clinical use. Further studies are needed to investigate the optimal use of macrolide antibiotics in patients with cancer and to explore the mechanisms underlying their effects on malignant progression.

## MATERIALS AND METHODS

### Ethics approval and consent to participate

All procedures performed in this study were carried out according to the principles of the Ethics Committee of Renmin Hospital of Wuhan University. The animal experiments were complied with the principles of the Animal Centre of the Renmin Hospital of Wuhan University.

### Meta-analysis

#### Search strategy and source

Relevant studies were searched from the Embase, PubMed and Web of Science databases from January, 2000 to October, 2021. The following terms were used for searching: “antibiotic,” “antibacterial agent,” “cancer,” “neoplasm,” “carcinoma” and “risk”. The referenced articles of the included publications were also reviewed.

#### Selection criteria

Two researchers (XY, ZJG) individually performed the search and assessment of all publications acquired.

The inclusion criteria were as follows:

- Case–control or cohort study.
- Original data comparing the risk of primary cancer between ever-users of macrolide antibiotics and nonuser were provided.
- Sufficient data to estimate risk estimates, including hazard ratios (HRs), risk ratios (RRs) and odds ratios (ORs), were available.

The exclusion criteria were as follows:

- *In-vitro* experiment, animal study, reviews, case-reports, cross-sectional studies, letters.
- Irrelevant exposure.
- Duplicate publications (the latest was selected).

#### Data extraction

The following data was extracted:

- study characteristics, including author, year, country, tumour type, sample size, and study setting and design.
- risk estimates, including HRs, RRs and ORs.

#### Quality assessment

Two researchers (XY, ZJG) independently conducted the quality assessment of the included studies. The Newcastle–Ottawa scale [62] was utilized to assess the quality of all studies included. A higher score indicated better methodological quality.

#### Statistical methods

The meta-analysis was conducted using the “meta” package of R software (version 4.0). Pooled risk estimates were calculated and displayed as RRs with corresponding 95% CIs. Heterogeneity among studies was assessed through the I^2^ test and Cochrane Q test [63] (P<0.1 or I^2^ >50% define substantial heterogeneity). Random-effects model was adapted to create forestplots if heterogeneity was present, otherwise, the fixed-effects model was adapted. Publication bias was evaluated through the Begg's test and Egger's test (p< 0.05 define substantial publication bias). Constancy of results was confirmed through sensitivity analysis.

### Cell culture and chemicals

GFP-Q74-expressing PC12 (Rat adrenal gland cells) cells were maintained in RPMI-1640 containing 10% horse serum and 5% fetal bovine serum (FBS) [64]. U2OS (human osteosarcoma cells) wild type (wt) and its variants with PERK-deficient (PERK−/−), TFEB and TFE3 double-knockout (*TFEB−/−TFE3−/−*), eIF2α mutant (S51A, non-phosphorylation eIF2α) or stably expressing GFP-LC3B, RFP-LC3B, GFP-ATG4-RFP-LC3BΔG3, mCherry-GFP-p62, GFP::CHOP, ATF6-GFP, TFEB-GFP or XBP1s-ΔDBD-venus and murine melanoma B16F10 cells and murine fibrosarcoma MCA205 cells were cultured in DMEM containing 10% FBS. Cell culture consumables were obtained from Life Technologies (Carlsbad, California, USA), and plastic materials were obtained from Corning (Corning, NY, USA) and Greiner Bio-One (Kremsmünster, Austria). U2OS cell lines with PERK-deficient (*PERK−/−*), TFEB and TFE3 double-knockout (*TFEB−/−TFE3−/−*), eIF2α mutant (S51A, non-phosphorylation eIF2α) and stably expressing GFP-LC3B, RFP-LC3B, GFP-ATG4-RFP-LC3BΔG3, mCherry-GFP-p62, LAMP1-GFP and GFP-LC3, GFP::CHOP, ATF6-GFP, GFP-TFEB or XBP1s-ΔDBD-venus were constructed in the past[29, 42, 65]. Macrolides were obtained from Target Mol (Boston, USA), Torin 1, Baf A1, TG and TM and NAC were from Sigma-Aldrich (Missouri, USA). Cells were treated with macrolides (40 µM), Torin 1 (300 nM), Baf A1 (100 nM), TG (3 μM), TM (3 μM) and NAC (3 mM).

### High-content microscopy

Cells were seeded in 384-well black imaging plates at a density of 2000 cells per well and allowed to adapt overnight. Cells were treated with the indicated agents 6 h for detecting the expression of GFP-LC3, RFP-LC3, Q74-GFP and GFP-TFEB, 6 h and 24 h for detecting the expression of CHOP::GFP, GFP-ATF6 and XBP1s-ΔDBD-venus, then fixed with 3.7% paraformaldehyde (Sigma-Aldrich) in PBS at 4°C overnight and then stained with 1 μg/ml Hoechst 33342 (Sigma-Aldrich), and examined using automated microscopy. Each experiment was analyzed at least in triplicate. Image acquisition was performed using an ImageXpress Micro XL automated microscope (Molecular Devices, Sunnyvale, California, USA) equipped with a 20 X PlanApo objective (Nikon, Tokyo, Japan). The MetaXpress software (Molecular Devices) was used to define and segment the cellular areas of interest, cytoplasm and nucleus, by excluding cellular debris and dead cells from the dataset. After normalization and statistical evaluation of the parameters of interest, the results are graphically depicted using R software. Finally, images are extracted and pixel intensities are scaled to be visible, ensuring that the same extent is used for all images in a given experiment.

### Immunofluorescence

Cells were treated with the indicated agents 6 h for detecting the expression of TFE3, CHOP, 6 h and 24 h for detecting the expression of PeIF2α, 6 h, 16 h and 24 h for detecting the expression of ATF4, then fixed with 3.7% paraformaldehyde (Sigma-Aldrich) in PBS at 4°C overnight and stained with 1 μg/ml Hoechst 33342 (Sigma-Aldrich), permeabilized with 0.1% Triton X100(#X100; Sigma-Aldrich), blocked with 5% bovine serum albumin (BSA) in PBS for 1 h, incubated with primary antibody at 4°C overnight, and then incubated with AlexaFluor conjugates 2 h at room temperature (RT; Thermo Fisher Scientific). At last, cells were imaged with automated fluorescence microscopy as described above. The antibodies used are listed in Table S3.

### Fluorescent LysoSensor

LysoSensor™ Green DND-189 from ThermoFisher Scientific was used to assess lysosome function in live cells. Cells were washed and stained with 5 μM LysoSensor™ Green DND-189 for 30 min in a 37°C incubator. Then cells were imaged using automated fluorescence microscopy as described above.

### Immunoblotting

As described previously [42], cells were washed twice with ice-cold PBS, and then collected with SDS loading buffer. After boiling for 10 min, the proteins were separated by SDS-PAGE, transferred to a nitrocellulose membrane, and detected with specific antibodies at 4 °C overnight, and then incubated with HRP-conjugated secondary antibody (CliniScience) for 2 h at RT. Antibodies are listed in Table S3.

### RNA isolation and Real-time PCR

Total RNA was extracted from samples using TRIzol (Pufei, Shanghai) according to the manufacturer's instructions. cDNA was synthesized with the Reverse Transcript Kit (Promega). Real-time PCR was performed with SYBR Green Master Mixture (TAKARA) on the Real-time PCR Detection System (Roche) in triplicate. Glyceraldehyde-3-phosphate dehydrogenase (GAPDH) was used as an endogenous normalization control. Quantification was based on the cycle threshold (Ct) value and calculated by the 2-ΔΔCt method. The sequences of the primers are listed in Table S4.

### Determination of ROS generation by flow cytometry

Intracellular ROS was measured by fluorescent dichlorofluorescein converted from cell permeable 2',7'-dichlorofluorescein diacetate (DCFH-DA, Beyotime, Shanghai, China) through oxidative conversion. Cells treated with AZD (40 µM, Sigma-Aldrich) with or without NAC (3 mM) and then incubated with DCFH-DA (10 μM, 20 min, 37°C). Finally, ROS production in cells was measured fluorometrically at wavelengths of 488nm for excitation and 525nm for emission (F-4000, HITACHI, Japan).

### Animal experimentation

Seven-week-old female wild-type C57BL/6 mice were maintained in a temperature-controlled and pathogen-free environment with 12-h light/dark cycles, with *ad libitum* access to food and water. Mice were given AZD (50 mg/kg, gavage) with or without NAC (100 mg/kg, intraperitoneal injection) every two days. For the B16F10: mice were injected subcutaneously with B16F10 cells (1*10^6^). These mice were sacrificed when any one of the tumor sizes reached 2000 mm^3^. For the MCA205: mice were injected subcutaneously with MCA205 cells (6*10^5^). Mice were sacrificed when tumor size reached 2000 mm^3^ or when signs of obvious discomfort were observed following the Institutional Animal Care and Use Committee of Renmin Hospital of Wuhan University.

### Immunohistochemistry (IHC)

Immunohistochemical staining was performed using an automatic staining machine (Leica Bond3). Following dehydration, antigenic epitopes were retrieved with a 10 mM citrate buffer and microwaving for 10 min. Specimens were then incubated with specific antibodies. Primary antibody staining was detected with peroxidase-conjugated IgG (1:500 diluted P0448, Dako, Glostrup, Denmark). Two independent pathologists evaluated the IHC staining results and scored them according to the percentage and intensity of positive tumor cells. The percentage of positive cells was scored as 0 < 10%, 1 = 10–20%, 2 = 21–50% and 3 > 50%. The staining intensity was evaluated as 0 = no staining, 1 = weak staining, 2 =moderate staining and 3 =strong staining. The final protein staining score was calculated by multiplying the percentage score by the intensity score.

### Statistical analysis

The data are reported as means ± SD of triplicate determinations, and experiments were repeated at least three times yielding similar results. Statistical significance between two groups was assessed by Student's t test. And the survival data was assessed by Log-rank test. Statistical analyses were performed with GraphPad Prism 8.0. P values <0.05 denote significance.

## SUPPLEMENTAL MATERIAL

Click here for supplemental data file.

All supplemental data for this article are available online at https://www.cell-stress.com/researcharticles/2023a-yu-cell-stress/.

## AUTHOR CONTRIBUTION

Concept and design: S.S., Y.T. and Q.W. Writing, review, and/or revision of the manuscript: X.Y., A.T., P.W., S.S., Y.T. and Q.W. Experimentation and analysis: X.Y., A.T., P.W., J.L., J.W., B.L., Z.L., S.L., Z.G. and S.S.

## Abbreviations:

AZD: – azithromycin;
Baf A1: – Bafilomycin A1;
CHOP: – C/EBP homologous protein;
eIF2α: – eukaryotic initiation Factor 2α;
EMT: – epithelial-mesenchymal transition;
ER: – endoplasmic reticulum;
GFP: – green fluorescent protein;
ISR: – integrated stress response;
NAC: – N-acetylcysteine;
PERK: – PKR-like ER kinase;
PKR: – protein kinase double-stranded RNA-dependent;
RA: – roxithromycin;
ROS: – reactive oxygen species;
SP: – spiramycin;
TFE3: – transcription factor E3;
TFEB: – transcription factor EB;
TG: – thapsigargin;
TM: – tunicamycin;
UPR: – unfolded protein response.
