# Supplementary material for: Macrolide antibiotics activate the integrated stress response and promote tumor proliferation
Source: Cell Stress. 2023 Mar 21;7(4):20–33. doi: 10.15698/cst2023.04.278 (PMC10069438; doi:10.15698/cst2023.04.278)
Supplement: Supplementary file 1 [file ces-07-020-s01.pdf]

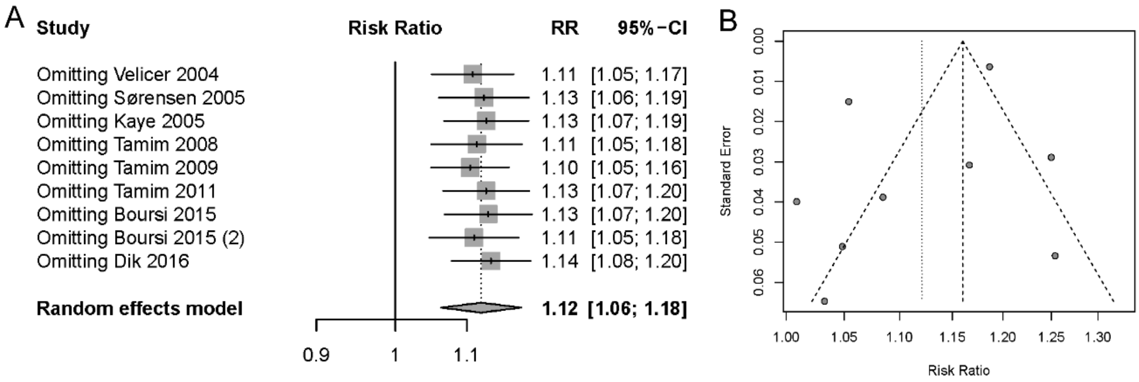

Supplementary Figure S1. (A) Influence analysis and **(B)** funnel plot in meta-analysis of macrolide antibiotic use and risk of malignant tumors.

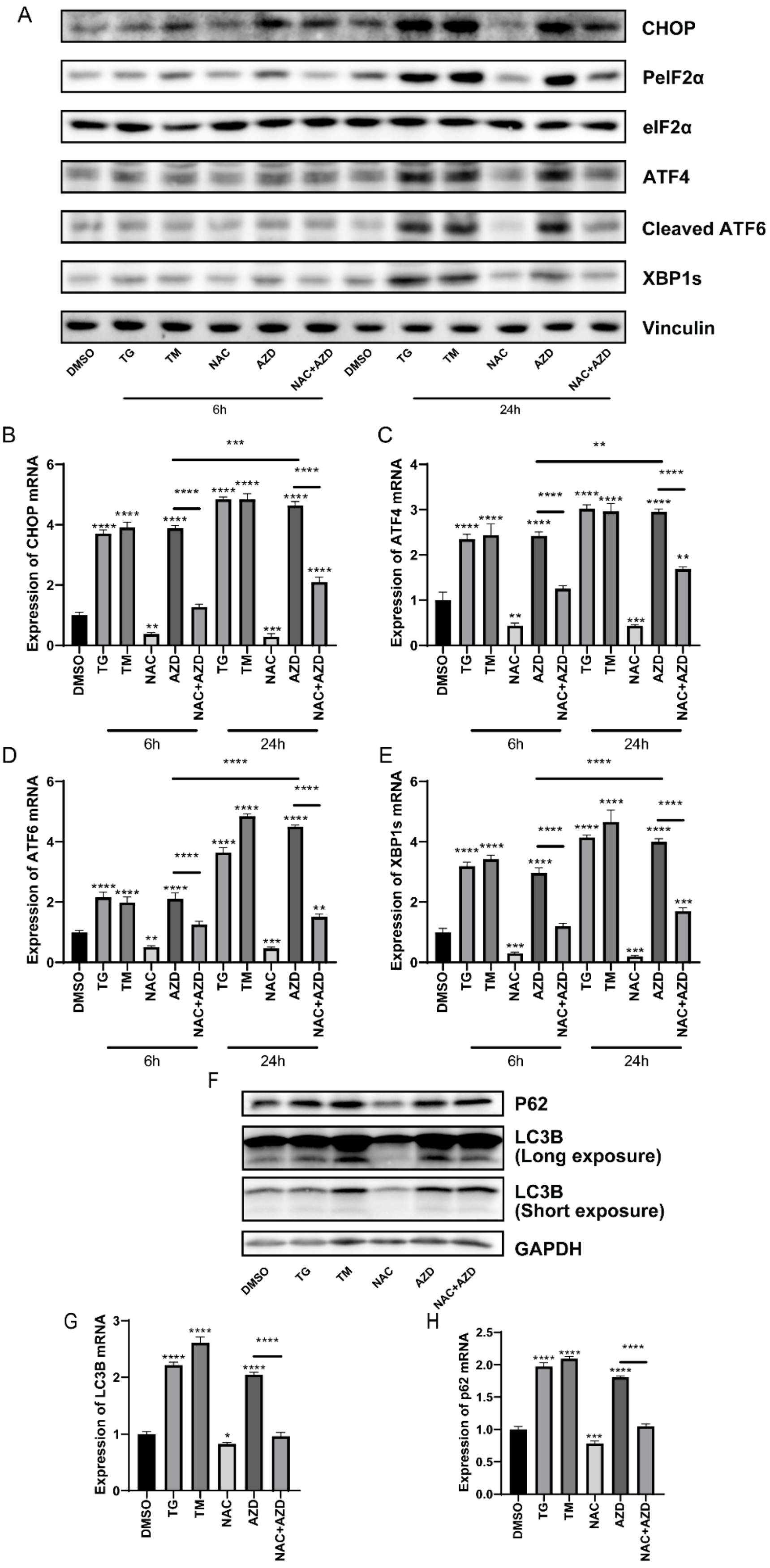

Supplementary Figure S2: AZD induces ROS accumulation and ER stress. **(A-E)** U2OS cells were treated with TM (3 μM), TG (3 μM), AZD (40 μM), NAC (3 mM) or NAC combined with AZD for 6 or 24 h. **(A)** The expression of CHOP, PeIF2α, ATF4, Cleaved ATF6 and XBP1s was assessed by Western Blot and the expression of mRNA of **(B)** CHOP, **(C)** ATF4, **(D)** ATF6 and **(E)** XBP1s was assessed by qtPCR. **(F-H)** U2OS cells were treated with TM, TG, AZD, NAC or NAC combined with AZD for 6. **(F)** The expression of LC3B and P62 was assessed by Western Blot and the expression of mRNA of **(G)** LC3B and **(H)** p62 was assessed by qtPCR. *P<0.05; **P < 0.01; ***P < 0.001; **** P < 0.0001 compared with DMSO/control. And the horizontal line indicates the comparison between the two groups.

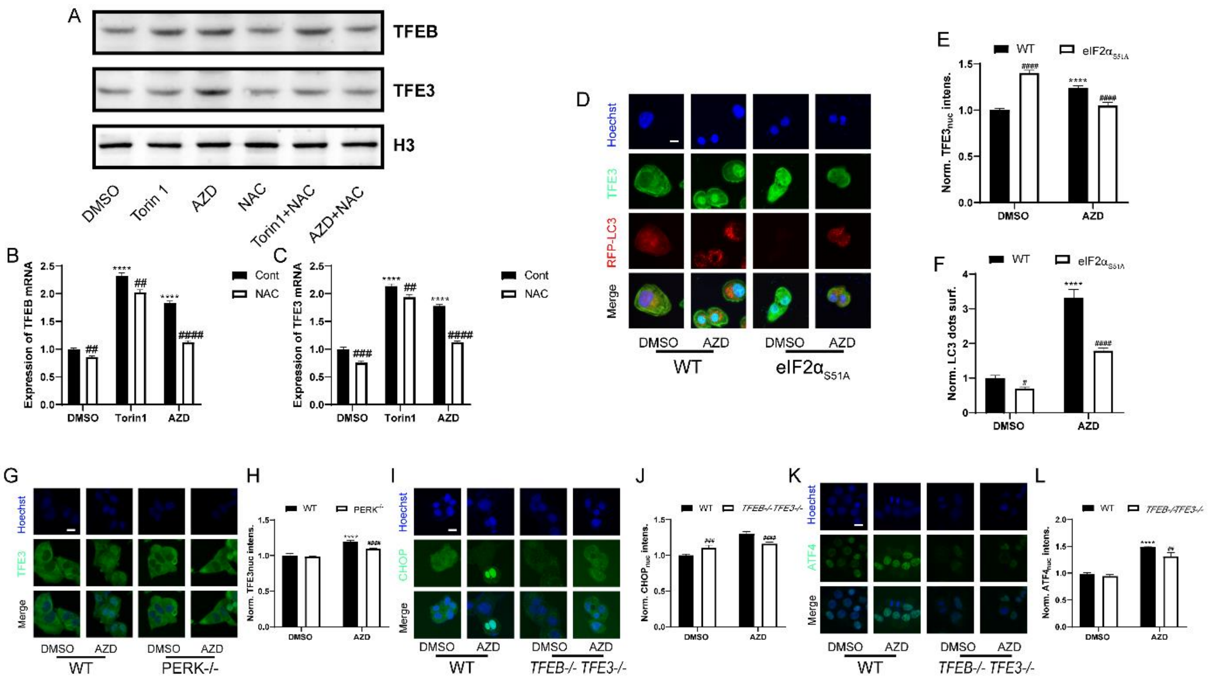

Supplementary Figure S3: AZD induced TFEB/TFE3 activation. (A-C) U2OS cells were treated with TM (3 μM), TG (3 μM), AZD (40 μM), NAC (3 mM) or NAC combined with AZD for 6 h. **(A)** The expression of TFEB and TFE3 in the nucleus was assessed by Western Blot and the expression of mRNA of **(B)** TFEB and **(C)** TFE3 was assessed by qtPCR. **(D-F)** RFP-LC3B-expression in U2OS with or without a mutant nonphosphorylation of eIF2α (eIF2αS51A) were treated with Torin 1 (300 nM) and AZD for 6 h, and TFE3 was assessed by immunostaining. The average nuclear intensity of TFE3 **(E)** and RFP-LC3B puncta **(F)** were assess. **(G, H)** U2OS cells with or without PERK were treated with AZD for 6 h. Representative images are presented **(G)**. The average nuclear intensity of TFE3 was assessed **(H)**. **(I-L)** U2OS cells with or without TFEB-/-TFE3-/- double-knockout were treated with AZD for assessing CHOP (24 h, **I**) and ATF4 (16 h, **K**), respectively. The average nuclear intensity was assessed in **(J)** and **(L)**. Scale bars equal 10 μm. (*P < 0.05, **P < 0.01, ***P < 0.001,****P<0.0001 vs. control; #P < 0.05, ##P < 0.01, ###P < 0.001, #### P < 0.0001 control or WT).

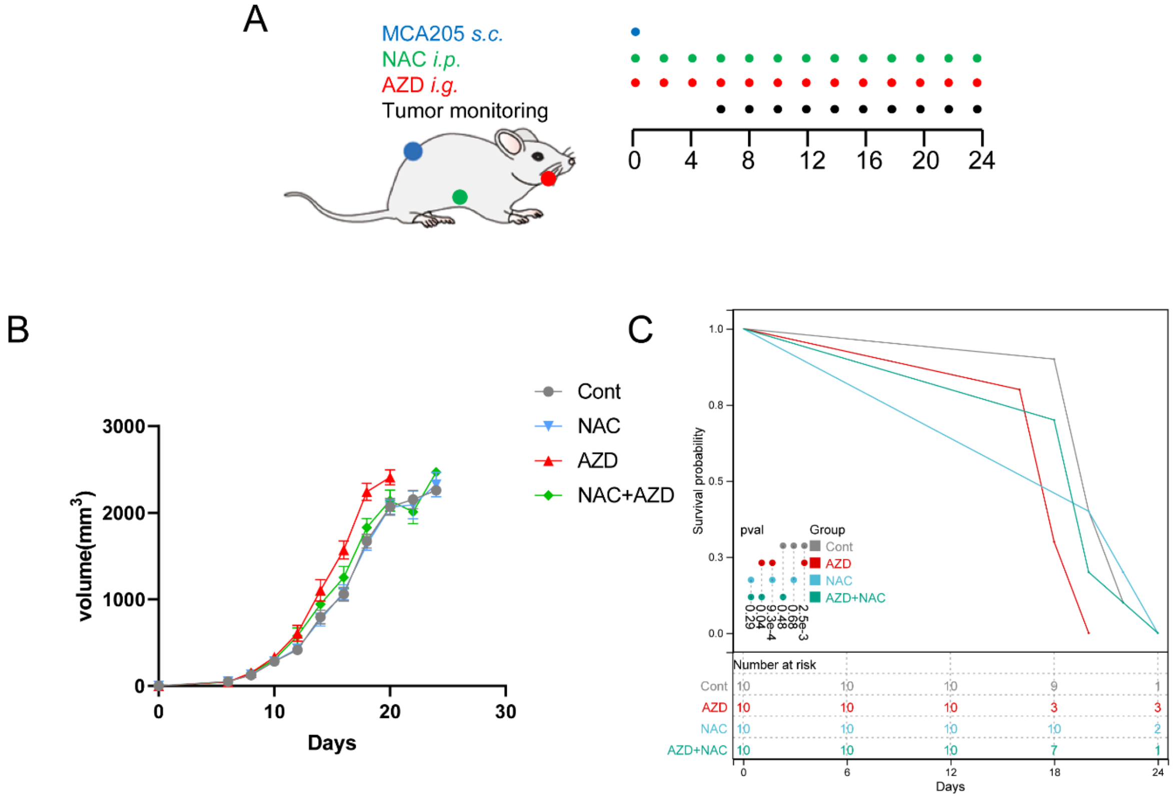

Supplementary Figure S4: **AZD induced ER stress and promoted tumor growth *in vivo*.** *In vivo* treatment of implanted murine MCA205 fibrosarcoma with administration of AZD combined or not with NAC (schematic view in A). **(B-C)** The data of administration of AZD combined or not with NAC, depicted as **(B)** growth curves (mean±SD), **(C)** survival curves.

Supplementary Table S1. Characteristics of all included studies.

First author and year	Ref.	Country	Cancer type	Number of macrolides		Number of control		Odd Ratios (95% CI)
				Event	Total	Event	Total	
Velicer et al, 2004	[32]	USA	Breast cancer	1934	8409	332	1810	1.33(1.17,1.51)
Sørensen et al, 2005	[33]	Denmark	Breast cancer	891	9272	1837	20736	1.09(1.01,1.19)
Kaye et al, 2005	[34]	UK	Breast cancer	245	1423	1023	6136	1.04(0.89,1.21)
Tamim et al, 2008	[35]	Canada	Breast cancer	1538	5442	1561	6443	1.23(1.14,1.34)
Tamim et al, 2010	[37]	Canada	Prostate cancer	1472	6350	2580	13910	1.33(1.23,1.42)
Tamim et al, 2011	[38]	Canada	Cervical cancer	85	478	107	482	0.76(0.55,1.04)
			Ovarian cancer	218	1037	227	1188	1.13(0.92,1.39)
			Uterine cancer	280	1328	308	1612	1.13(0.94,1.36)
Boursi et al, 2015a	[39]	UK	Colorectal cancer	4388	20661	16602	82383	1.07(1.03,1.11)
Boursi et al, 2015b	[40]	UK	Breast cancer	5715	23346	25637	131291	1.34(1.29,1.38)
			Lung cancer	4435	16477	14708	77139	1.56(1.5,1.63)
			Esophagus cancer	1081	4887	5027	25071	1.13(1.05,1.22)
			Gastric cancer	714	3095	3145	15786	1.21(1.1,1.32)
			Hepatocellular cancer	248	1010	1051	5344	1.33(1.13,1.56)
			Biliary cancer	201	850	709	3603	1.26(1.06,1.51)
			Gallbladder cancer	76	358	289	1433	1.07(0.8,1.42)
			Pancreas cancer	761	3443	3352	16742	1.13(1.04,1.24)
			Prostate cancer	4348	2012	22864	113040	1.09(1.05,1.13)
			Renal cancer	301	1238	1246	6375	1.32(1.15,1.53)
			Bladder cancer	2321	10340	11119	55521	1.16(1.1,1.22)
			Melanoma cancer	1473	7090	7753	38482	1.04(0.98,1.11)
			Cervical cancer	519	2449	2875	14361	1.07(0.97,1.19)
			Osteosarcoma	59	285	294	1448	1.02(0.75,1.4)
			Multiple myeloma	629	2505	2491	12830	1.39(1.26,1.54)
Dik et al, 2016	[41]	Netherlands	Colorectal cancer	583	2875	3446	17142	1.01(0.92,1.12)

Supplementary Table S2. Human osteosarcoma U2OS and neuroglioma H4 cells stably expressing GFP-LC3 or RFP-LC3 were treated with the autophagy inducer torin 1 (300 nM), the inhibitor of autophagic flux bafilomycin A1 (Baf A1; 100 nM) or macrolides (40 µM) for 6 h.

Agent	GFP-LC3 positive puncta	Q74-GFP positive puncta
DMSO	1	1
Torin 1	2.578	0.687
Bra A1	2.024	2.437
Erythromycin	1.189	1.943
Oleandomycin	1.351	1.797
Clarithromycin	2.193	1.821
Roxithromycin	1.794	1.417
Dirithromycin	1.033	2.614
Telithromycin	1.716	2.759
Cethromycin	1.696	1.629
Flurithromycin	2.16	2.01
Erythromycin ethylsuccinate	1.167	2.632
Erythromycin estolate	1.464	2.024
Azithromycin	1.379	1.906
Midecamycin	1.147	1.394
Midecamycin Acetate	1.169	1.618
kitasamycin	1.291	0.866
Acetylkitasamycin	2.359	1.832
Josamycin	2.109	1.47
Spiramycin	2.012	2.866
Acetylspiramycin/Spiramycin II	4.233	1.816
Rokitamycin	1.287	1.316
Tylosin	1.207	1.198
Rosaramicin	1.741	2.591
Tilmicosin	2.013	1.544
Kitasamycin tartrate	2.444	2.425
Amphotericin B	1.3	1.811
Pentamycin	2.539	2.206
Fidaxomicin	1.419	2.89
Ascomycin	1.379	2.098
Tacrolimus	1.349	0.751
Pimecrolimus	1.122	0.712
Everolimus	1.937	0.537
Sirolimus/Rapamycin	2	0.803

Abbreviation: Bra A1: bafilomycin A1

Supplementary Table S3. Antibodies used in this work.

Antibodies	Source	Identifier	Application	Dilution
PeIF2α	Abcam	#ab32157	Immunofluorescence<br>Immunoblotting<br>Immunohistochemistry	1:1000<br>1:2000<br>1:100
TFE3	Abcam	#ab93808	Immunofluorescence<br>Immunoblotting	1:400<br>1:4000
ATF4	Abcam	#ab23760	Immunofluorescence<br>Immunoblotting	1:1000<br>1:5000
β-actin	Abcam	#ab6276	Immunoblotting	1:7000
CHOP	Cell Signaling Technology	#2895	Immunoblotting<br>Immunofluorescence	1:1000<br>1:800
LC3B	Cell Signaling Technology	#83506	Immunoblotting<br>Immunofluorescence<br>Immunohistochemistry	1:1000<br>1:200<br>1:100
p62	Cell Signaling Technology	#88588	Immunoblotting	1:1000
TFEB	Cell Signaling Technology	#4240	Immunoblotting	1:1000
GAPDH	Cell Signaling Technology	#2118	Immunoblotting	1:1000
H3	Cell Signaling Technology	#9715	Immunoblotting	1:1000
ATF6	Proteintech	#24169-1-AP	Immunoblotting	1:4000
XBP1s	Proteintech	#24868-1-AP	Immunoblotting	1:2000
eIF2α	Proteintech	#11170-1-AP	Immunoblotting	1:10000
Tubulin	Proteintech	#11224-1-AP	Immunoblotting	1:4000
Vinculin	Proteintech	# 66305-1-Ig	Immunoblotting	1:5000

Supplementary Table S4. Sequences of the primers used in this work.

Genes	GenBank Accession	Forward Sequence	Reverse Sequence
CHOP	NM_001195053.1	TTCTCTGGCTTGGCTGACTG	TCCTCCTCTTCCTCCTGAGC
ATF4	NM_001675.4	GGGAAGCGATTTAACGAGCG	TCTTGGTTCCTGCCACGTTT
ATF6	NM_001410890.1	ACCACTAGTAGTATCAGGAACTCAG	AATGTGTCTCCCCTTCTGCG
XBP1s	NM_001079539.2	AGCTTTTACGAGAGAAAACTCA	GCCTGCACCTGCTGCG
TFEB	NM_001167827.3	TCCAACAAGGGAAGGTGACAT	CAGCCTGAGCTTGCTGTCAT
TFB3	NM_001282142.2	CTTCGCTCAAGGGGGCTC	AGACGCCAACCACAGAGATG
LC3B	NM_022818.5	TTCAGGTTCACAAAACCCGC	TCTCACACAGCCCGTTTACC
P62	NM_001142298.2	CATTGCGGAGCCTCATCTCC	TCCTCGTCACTGGAAAAGGC
GAPDH	NM_ 007073	TGACTTCAACAGCGACACCCA	CACCCTGTTGCTGTAGCCAAA

REFERENCES

32. Velicer CM, Heckbert SR, Lampe JW, Potter JD, Robertson CA, Taplin SH (**2004**). Antibiotic use in relation to the risk of breast cancer. **JAMA** 291(7): 827-835. doi: 10.1001/jama.291.7.827

33. Sørensen HT, Skriver MV, Friis S, McLaughlin JK, Blot WJ, Baron JA (**2005**). Use of antibiotics and risk of breast cancer: a population-based case-control study. **Br J Cancer** 92(3): 594-596. doi: 10.1038/sj.bjc.6602313

34. Kaye JA, Jick H (**2005**). Antibiotics and the risk of breast cancer. **Epidemiology** 16(5): 688-690. doi: 10.1097/01.ede.0000172131.84877.42

35. Tamim HM, Hanley JA, Hajeer AH, Boivin JF, Collet JP (**2008**). Risk of breast cancer in relation to antibiotic use. **Pharmacoepidemiol Drug Saf** 17(2): 144-150. doi: 10.1002/pds.1512

37. Tamim HM, Hajeer AH, Boivin JF, Collet JP (**2010**). Association between antibiotic use and risk of prostate cancer. **Int J Cancer** 127(4): 952-960. doi: 10.1002/ijc.25139

38. Tamim HM, Musallam KM, Al Kadri HM, Boivin JF, Collet JP (**2011**). Antibiotic use and risk of gynecological cancer. **Eur J Obstet Gynecol Reprod Biol** 159(2): 388-393. doi: 10.1016/j.ejogrb.2011.06.018

39. Boursi B, Haynes K, Mamtani R, Yang YX (**2015**). Impact of antibiotic exposure on the risk of colorectal cancer. **Pharmacoepidemiol Drug Saf** 24(5): 534-542. doi: 10.1002/pds.3765

40. Boursi B, Mamtani R, Haynes K, Yang YX (**2015**). Recurrent antibiotic exposure may promote cancer formation--Another step in understanding the role of the human microbiota? **Eur J Cancer** 51(17): 2655-2664. doi: 10.1016/j.ejca.2015.08.015

41. Dik VK, van Oijen MG, Smeets HM, Siersema PD (**2016**). Frequent Use of Antibiotics Is Associated with Colorectal Cancer Risk: Results of a Nested Case-Control Study. **Dig Dis Sci** 61(1): 255-264. doi: 10.1007/s10620-015-3828-0
